# What Strain Analysis Adds to Diagnosis and Prognosis in Heart Failure Patients

**DOI:** 10.3390/jcm12030836

**Published:** 2023-01-20

**Authors:** Guido Pastorini, Fabio Anastasio, Mauro Feola

**Affiliations:** Division of Cardiology, Ospedale Regina Montis Regalis Mondovì, ASL CN1, 12084 Cuneo, Italy

**Keywords:** heart failure, echocardiography, global longitudinal strain

## Abstract

Heart failure (HF) is a common disease that requires appropriate tools to correctly predict cardiovascular outcomes. Echocardiography represents the most commonly used method for assessing left ventricular ejection fraction and a cornerstone in the detection of HF, but it fails to procure an optimal level of inter-observer variability, leading to unsatisfactory prediction of cardiovascular outcomes. In this review, we discuss emerging clinical tools (global longitudinal strain of the left ventricle, the right ventricle, and the left atrium) that permitted an improvement in the diagnosis and ameliorated the risk stratification across different HF phenotypes. The review analyzes the speckle-tracking contributions to the field, discussing the limitations and advantages in clinical practice.

## 1. Background

Heart failure (HF) is a pathophysiological condition with signs and symptoms due to abnormal cardiac function, resulting in the inability of the heart to pump the blood required to meet the needs of metabolic tissues, or due to the high left ventricular filling pressure, despite preserved contraction. According to the European guidelines, HF is classified based on the left ventricular (LV) systolic function: HF with preserved ejection fraction (HFpEF) (ejection fraction (EF) > 50%), HF with mid-range EF (HFmEF) (EF 40–49%), HF with reduced EF (HFrEF) (EF < 40%), and improved EF (HFimpEF) with a baseline EF ≤ 40% and a subsequent improvement of at least 10% from the baseline EF [1].

Despite this classification, the prognosis is similar between the LVEF groups [2], which underlies the limited role of EF measured by echocardiography. In fact, LVEF measurement, the most acknowledged parameter, depends on the preload and the afterload with a high intra- and inter-observer variability, affecting reproducibility, as well as operators’ expertise [3,4]. In addition, the analysis of LVEF is limited by the inability to represent segmental abnormalities of myocardial contractility in patients with normal EF due to a compensatory mechanism in other regions. Furthermore, relying solely on geometric assumptions to diagnose the presence of HF appears to be limited, especially when HFpEF becomes the dominant phenotype [5]. This condition generally affects patients with hypertrophic cardiomyopathy (HCM), ischemic heart disease, and diabetes mellitus, in which, due to geometric confounders, LVEF measurement is incapable of detecting initial impairment of contraction. The initial alteration, after myocardial injury, is a reduction of longitudinal contraction, despite a normal LVEF, resulting in an abnormal neurohumoral activation, and a subsequent loop of deleterious effects on the cardiovascular system, which could explain the similar prognostic values in different HF patients [6,7,8]. To overcome these limitations, new methods have been implemented in daily practice, such as the analysis of longitudinal deformation, that expresses longitudinal shortening, as a percentage. In the search for better prognostic markers, longitudinal shortening of the left atrium (LA) and right ventricle (RV) was also examined for better patient stratification.

## 2. Global Longitudinal Strain

**Figure 1:**
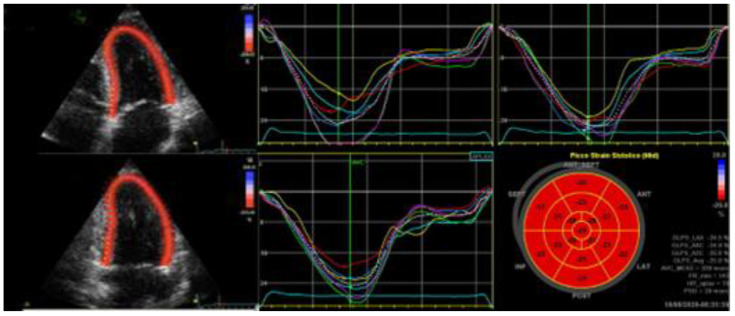
Two-dimensional speckle tracking: from recorded multiple heart beats during breath-hold, an apical four-chamber view, an apical two-chamber view, and an apical three-chamber view have been stored. Once the specific views have been named, it is required to trace the endocardial border in a still frame, setting the ROI. Finally, the evaluation of the deformation graphs and values may be calculated. The segmental peak systolic strains are represented in bullseye. ROI indicates region of interest.

Analysis of longitudinal deformation could detect subclinical dysfunction and identify the initial alteration of LV myocardial contraction. This analysis could be performed using strain-based imaging techniques (speckle-tracking echocardiography): an operator-friendly, less angle-dependent, post-processing computer algorithm that measures the deformation of the left ventricle in the longitudinal, circumferential, and radial planes [9]. During a two-dimensional echocardiogram, a region of interest on the myocardial wall is traced in the apical view. Myocardial footprints were identified and tracked during systole and diastole; their changes in length were subsequently analyzed and processed using the following formula:

(1) $$\varepsilon =\frac{\left(L-L_{0}\right)}{L_{0}}$$

where ε indicates strain, L indicates the length after deformation, and L_0_ indicates the baseline length. It is important to consider the normal values of the left ventricular global longitudinal strain (GLS) in terms of clinical applications which varied form −15.9% to −22.1% [9] (Figure 1).

Moreover, GLS analysis could be applied to the RV, with a better diagnostic capability compared to standard echo measures, and the LA, in order to obtain additional diagnostic information regarding diastolic function, through the analysis of atrial strain [10].

These approaches are, however, far from faultless, and there are important limitations that need to be acknowledged, such as the absence of large-scale, independent randomized studies. GLS may be influenced and flawed by geometric assumptions, poor acoustic windows, inappropriate measures due to imprecise customization, or inaccurate tracking of the LV myocardium throughout systole. Moreover, GLS may have different values depending on the age, sex, and loading conditions. There are small differences between different vendors; subsequently improved software versions were released to improve the inter-vendor agreement [11].

Nonetheless, GLS can be a useful clinical tool to improve risk stratification in patients with HF, as this parameter correlates with the severity of HF, and it can help identify different cardiomyopathy phenotypes suggesting disease causes and severity.

### 2.1. Recognition of the Different HF Phenotypes

#### 2.1.1. HFrEF

GLS demonstrated to be superior to LVEF in assessing the likelihood of all-cause mortality in patients with acute congestive HF, regardless of clinical characteristics and systolic function [12]. This method has been validated in different HF phenotypes and clinical conditions [13,14,15,16], and the superiority of the prognostic values of GLS over LVEF was confirmed in more than 4000 patients with acute HF, in which, over a period of 8 years, GLS showed an inverse correlation with mortality (whereas LVEF failed for the same patients), and an increased risk of death by 5% every 1% decrease in LV-GLS [17]. Accordingly, the European Society of Cardiology HF Association (HFA) recommends including this parameter to improve the diagnostic accuracy in patients with HF [18].

The GLS enables the evaluation of mechanical dispersion, namely the peak strain dispersion (PSD), which represents the contractile dispersion defined as the standard deviation of the contractile duration compared to the regional strain curves. PSD accurately reflects the dyssynchrony between different LV regions, functional heterogeneity of contraction, and effective LV global work [19].

PSD proved to be a good predictor of ventricular arrhythmias independent of LVEF [20]. Similarly, an absolute LV-GLS value of <8.3%, before CRT implantation, was correlated with fatal events, demonstrating a close relationship with the presence of transmural scars, using cardiac magnetic resonance [20]. Moreover, in CRT non-responder patients, the loss of volumetric response could also be explained by a lack of improvement in LV-GLS and PSD values [21].

This feature appeared to be beneficial in patients with ischemic heart disease and HF-identifying patients, who could most benefit from implantable cardioverter–defibrillator (ICD) implantation. However, this has not been confirmed in any independent, randomized, large study [19].

RV systolic dysfunction remains an important predictor of major events in patients with congestive HF [22]. The quantification of RV volumes could be difficult, especially with the standard transthoracic echocardiography, due to the complex geometry and retrosternal location. In clinical practice, RV function is then usually assessed through a small portion of the right ventricle, lateral annulus movement, using linear functional measures, such as TAPSE and TDI. For a more comprehensive assessment of RV performance, RV-GLS can be used, especially with RV-GLS (including the interventricular septum in the ROI) and RV-free wall strain (focusing only on the free wall), resulting in a good predictor of adverse cardiovascular events when diminished [23]. When both the LV and the RV strains are assessed, incremental prognostic information is provided. In more than 600 patients admitted for acute HF with a median follow-up of 427 days, the inclusion of RV-GLS to conventional echocardiographic measures markedly improved prognostic significance. The patients with worse RV-GLS function experienced unfavorable outcomes [24].

#### 2.1.2. HFpEF

A preserved EF does not guarantee the absence of clinical HF. Almost half of the patients that presented with signs and symptoms of HF displayed a preserved EF on the echocardiogram [25]. Conversely, GLS has the advantage of early detection of cardiac dysfunction, providing a more objective and complementary method for assessing myocardial function. More than 90% of patients with HFpEF showed a decreased LV-GLS, with a median of 15.2% [17]. This was already confirmed in a sub-study of the TOPCAT trial, in which patients with HFpEF had lower LV-GLS values than individuals without HF [26]. Moreover, the degree of reduction in LV-GLS is likely to be clinically significant, showing worse prognosis in the more impaired scores [25]. While in earlier studies it was emphasized that HFpEF could be considered a synonym of diastolic dysfunction, moderate-to-severe diastolic dysfunction is present only in 44% of the population, despite a lack of a uniform and reliable definition of diastolic dysfunction in epidemiological studies [27,28]. Furthermore, in a dedicated echocardiographic CHARM sub-study, 28% of randomly selected residents had diastolic dysfunction, whereas only 2.2% had HF [27]. The breakthrough of LV-GLS renewed our knowledge of the physiopathology of HFpEF, showing the presence of both systolic and diastolic dysfunction [29]. Impaired release and uptake of intracellular calcium have been suggested as an underlying mechanism in these patients [29]. In effect, a plethora of studies highlight that a preserved EF may coexist with a significant alteration in longitudinal systolic, along with diastolic, function in patients with ischemic heart disease, hypertension, or diabetes [30,31,32]. This reduction is associated with the gradual expansion of myocardial fibrosis [31].

**Figure 2:**
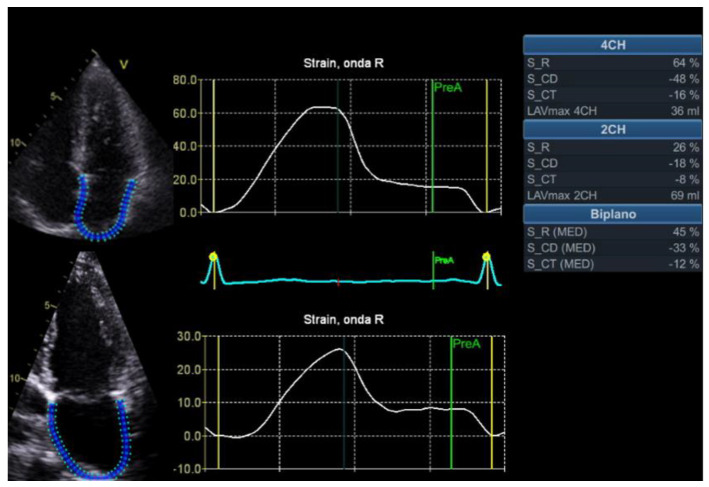
Example calculation of LA strain, with corresponding LA contractile, conduit and reservoir functions, respectively. LA indicates left atrium.

The diagnosis of HFpEF can also be improved by assessing LA function. Strain analysis can be applied to the LA providing an accurate estimation of the LV filling pressure and the LA function. All phases of the LA loop, reservoir, conduit, and booster pump were analyzed. It was detected using 2D speckle-tracking echocardiography, placing a region of interest (ROI) on the LA in Four- and two-chamber views (Figure 2). Traditionally, diastolic function has been evaluated through mitral early diastolic inflow velocity (E), early diastolic mitral annular velocity (e’), the respective ratio, the index volume of the LA, and the peak velocity of tricuspid regurgitation. The addition of LA strain increases the sensitivity of increased LV filling pressure through early recognition of alteration, less dependence on loading conditions [33], and higher reproducibility and feasibility [34]. Furthermore, LA strain seems to be less dependent on preload, index, LV mass, LA volume and afterload [35].

When invasive LV filling pressures were evaluated, LA strain correlated better than standard parameters; the existing data showed only a modest correlation between E/e’ and invasive filling pressures [36]. Abnormal LA strain, more commonly than increased LA volume, was also correlated with worse symptoms and more hospitalization due to HF, suggesting that an early detection of worsening LA strain value could improve the prevention of future outcomes, even in HFpEF patients [37]. Furthermore, in this population, reduced LA strain was associated with impaired exercise capacity, as well as elevated E/e’ ratio [38], and a higher risk of HF hospitalization. It seems that impaired contractile function and elastic recoil of the LA, measured through LA strain, due to the progressive increase in LV filling pressure and fibrotic remodeling, may lead to HFpEF symptoms and increased dependency on the LA pump for appropriate LV filling [39]. In addition, analyzing LA reservoir strain, an absolute value of 23% or less was associated with poor exercise capacity and worse prognosis [40]. According to expert consensus documents, although a universal standard validation is still missing, it has been suggested that the LA reservoir strain <18% can be included in the estimation of the LV filling pressure [41].

Increasing evidence indeed suggests that the LA remodeling is distinct from the LA enlargement because it involves LA changes, such as fibrosis, even before atrial enlargement [42].

Despite these promising results, LA strain has some important limitations: a lack of generally accepted standard validation, a standardized methodology, limited software distribution, and it is a preload-dependent method, even if to a lesser degree than LA volume, which makes the interpretation of the atrial function index more difficult due to the interaction between atrial and ventricular functions [43].

This method needs to be validated in randomized trials, and the correlation between LA strain and LV filling needs to be evaluated in a plethora of different scenarios, e.g., restrictive cardiomyopathy, mitral regurgitation, HCM, patients suffering from hyperdynamic states.

#### 2.1.3. HFmEF

HFmEF is defined as the presence of typical HF symptoms and an EF of 41–49%, encompassing 3–24% of patients suffering from HF [44]. The novel classification of HFmEF remains controversial, since traditional methods cannot predict exactly which patients are at risk of developing HfrEF and poorer outcomes. Despite this, patients diagnosed with HfmEF are believed to have a prognosis and clinical presentation similar to those diagnosed with HfpEF [45]. GLS appears to be a more sensitive marker of systolic dysfunction than the other methods used in clinical practice. When the impact of reduced GLS in patients hospitalized for HmEF was evaluated, a GLS absolute value < 13 appeared to be significantly related to increased 12-month mortality and 12-month cardiovascular events. After adjusting for other important prognostic markers, such as chronic kidney disease, pulmonary disease, and diabetes, GLS was significantly associated with increased mortality and cardiovascular events [46].

Within the HFmEF spectrum, there is another clinical entity, distinct from HFrEF and HFpEF: HFimpEF.

Several subsequent studies have identified HF patients with improved EF (HFimpEF) as the new clinical entity, distinct from HFrEF and HFpEF. HF with a second measurement of LVEF > 40% and a ≥10% increase from the baseline LVEF of ≤40% is defined as HFimpEF [2]. Previous HFrEF patients who developed HFimpEF during follow-up not only had a better prognosis, but also had a significant improvement in health-related quality of life [47], with a reduction of 56% in mortality and 60% in cardiac hospitalization, compared to HFrEF [48]. However, their clinical course is variable and there is a risk of future events, such as EF deterioration. In fact, there was an association between absolute GLS scores and outcomes, independent of EF: each point decrease in absolute GLS was associated with a higher risk of the composite endpoint (cardiovascular mortality or HF hospitalization/emergency treatment), particularly when absolute GLS was lower than 12.7% [49]. However, larger studies are warranted to validate these findings to consolidate the role of GLS in patients with HfmEF, and have a universally acknowledged parameter.

#### 2.1.4. Hypertrophic Cardiomyopathy

A typical conundrum during clinical practice could be to differentiate between physiological characteristics, such as in the athlete’s heart, and left ventricular hypertrophy (LVH), a hallmark frequently described in patients with HFpEF. LV-GLS may be useful because there are typical regional alterations distinctive to specific diseases.

HCM is characterized by symptoms typically attributed to HF, and is associated with a normal EF (except during the later stage) and an impaired LV-GLS. Given the several subtypes of HCM, characteristic regional alterations have been described, such as reduction in the septal segments in sigmoid HCM, or by a diffuse reduction in the apical segments, with global reduction of an absolute LV-GLS (15.1% versus 20.3%) [50]. After adjustments for myocardial thickness, regional strain was significantly reduced, especially in the most severely hypertrophic patients [51]. It is important to additionally highlight the implications of these values: decreased LV-GLS in these patients is correlated to myocardial fibrosis identified using magnetic resonance and subsequently to clinical adverse outcomes, such as all causes of deaths and ventricular arrhythmia [52,53].

PSD seems to be especially useful in identifying patients at risk of ventricular arrhythmia. A >67 ms increase in PSD in patients with HCM was significantly associated with a future risk of ventricular tachycardia [54].

These patients also usually experience paroxysmal atrial fibrillation and left atrial structural remodeling, thus putting them at an increased risk of adverse cardiovascular outcomes. It is pivotal to consider any modifications that could affect the LA function, and therefore LA strain, allowing for the categorization of the LV diastolic function and the forecasting of HF events. In 104 HCM patients, the best cutoffs for poor cardiovascular outcomes (comprising heart failure, stroke, and death) were absolute values < 23.8% for reservoir strain, and <10.2% for conduit strain [55]. In another study with 414 patients, low LA strain (<24%) was associated with worse HF-free survival and had incremental prognostic values for incident HF events compared to traditional echocardiographic parameters [56].

However, attention should be paid to possible confounders, such as Fabry cardiomyopathy (FC), a disorder caused by deficient enzyme activity of α-galactosidase A (α-Gal A): in affected individuals, the principal enzyme substrate, globotriaosylceramide (Gb3), accumulates in cytoplasmic lysosome, leading to organ dysfunction and symptoms [57].

Cardiac involvement in Fabry disease has multiple manifestations, including HF and progressive LVH. It is therefore recommended to be included in the differential diagnosis of adults with unexplained LVH [58].

Strain analysis has been shown to be a useful tool in the detection of systolic dysfunction in patients with FC, especially for early detection, as reduced GLS (mean absolute value < 16.5%) may be a marker of early FC [59]. However, when LVH is present, it is also important to consider possible regional strain pattern: rather than focusing solely on the mean GLS values, evaluation of individual wall segments may aid in the differential diagnosis. It is important to consider that even though the overall average strain value may appear normal, certain areas have strain abnormalities—especially the basal and mid-inferior segments. These areas are more likely to have abnormal absolute low GLS values compared to other regions with significant discrepancies. In addition, increased interventricular septal and left ventricular posterior wall thickness are correlated with greater strain abnormalities. Moreover, strain imaging can detect early evidence of cardiomyopathy, even before changes can be fully detected by an MRI [60].

#### 2.1.5. Restrictive Cardiomyopathies: Cardiac Amyloidosis and Cardiac Sarcoidosis

**Figure 3:**
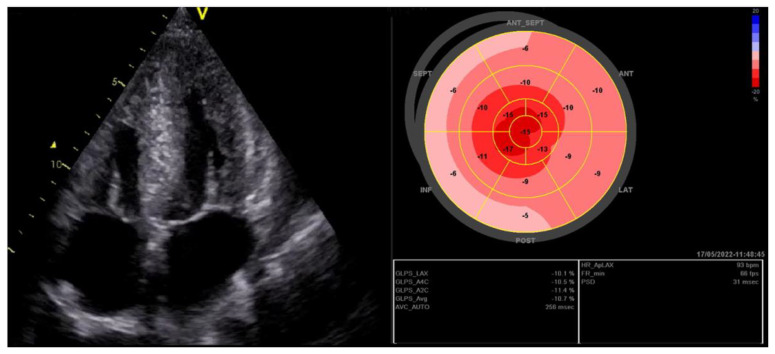
In the presence of left ventricular hypertrophy, speckle tracking could be an added value, showing a severe impairment of peak systolic longitudinal strain values (with the typical pattern of amyloidosis). LVH indicates left ventricular hypertrophy.

Cardiac amyloidosis is another cause of HFpEF, often diagnosed late. When this condition is suspected (i.e., in the presence of signs and symptoms of HF, abnormal hypertrophy, abnormal diastole), LV-GLS could improve the diagnosis through the detection of specific patterns, such as the “apical sparing” pattern, characterized by preserved LV-GLS values on an apical region, along with reduced values in the basal segment, showing a high discriminatory power, with a sensitivity of 93% and specificity of 82%, between these two conditions from controls [61] (Figure 3). Moreover, a similar cardiac involvement with an apical sparing pattern could be found in patients with Alzheimer’s disease, suggesting a likely association between Aβ amyloid deposition and myocardial involvement [62].

However, it could be challenging to differentiate between hypertensive heart disease and cardiac amyloidosis because both share an increased left ventricular wall thickness. In one study, when the LA function was assessed, LA strain in confirmed cases of cardiac amyloidosis was significantly reduced compared to the control group of hypertensive patients, irrespective of amyloid light chain or amyloid transthyretin subgroup. Despite similar LV wall thicknesses, a reservoir strain cut-off value of 20% was 86.4% sensitive and 88.6% specific for identifying cardiac amyloidosis in this cohort [63].

Sarcoidosis is a systemic granulomatous disease that can involve the myocardium [64]. Sudden cardiac death and progressive HF are known possible complications in these patients, even in those without significant echocardiographic abnormalities [65,66]. Specifically, the incidence of HF in sarcoidosis, although low, is more than double that of the general population, especially after diagnosis [67]. Therefore, efforts are needed to improve the low (25%) sensitivity of conventional 2D echocardiography to detect cardiac sarcoidosis. In a cohort of 31 sarcoidosis patients, an absolute GLS cutoff of 17% had high sensitivity and specificity for early detection, and the reduction in GLS size was inversely related to LGE burden on the cardiac MRI [68]. In another study with 83 patients, when evaluating individual wall segments, the lowest mean values were found in the LV basal and mid-interventricular inferoseptum, as well as in the inferior wall. It was also shown that the patients with impaired left ventricular GLS (absolute average value < 14%) had significantly higher rates of hospitalization and HF [69]. This value is consistent when another cohort of 117 patients with sarcoidosis was evaluated and followed-up for an average of 57 months: GLS absolute value < 13.6% was considered more associated with adverse outcomes, such as HF related hospitalizations, even after adjustment for multiple potential confounders [70].

#### 2.1.6. Cardiotoxicity

The role of LV-GLS in identifying early subclinical myocardial injury has become central in cardio-oncology, although it is not effectively used clinically. Early identification of adverse effects on myocardial tissue is pivotal to improve the effectiveness of chemotherapy and to adjust the correct cardiovascular therapy as soon as possible, to avoid further deterioration. The superiority of LV-GLS over LVEF, in these patients, was confirmed in a trial of patients who underwent anthracycline therapy [71]. After one year of follow-up, the control group guided by surveillance of EF only showed a larger EF reduction than the LV-GLS surveillance group due to the delayed diagnosis of myocardial injury. This resulted in the absence of medical interventions, such as chemotherapy discontinuation, and initiation of therapy with angiotensin-converting enzyme inhibitors and beta-blockers [71]. These findings have contributed to incorporate strain assessment during echocardiography laboratory protocols of the principal societies of imaging. In these patients, an absolute LV-GLS value < 15% or a reduction from the baseline value is considered a hallmark of early systolic dysfunction [72].

### 2.2. RV Failure

RV systolic dysfunction remains an important predictor of major events in patients with HF [22]. The quantification of RV volumes could be complicated, especially with standard transthoracic echocardiography, due to the complex geometry and retrosternal location. In clinical practice, RV function is then usually assessed through a small portion of the right ventricle (lateral annulus movement) using linear functional measures, such as TAPSE and TDI. For a more comprehensive assessment of RV performance, RV-GLS can be used, especially with RV-GLS (including the interventricular septum in the ROI) and RV-free wall strain (focusing only on the free wall), resulting in a good predictor of adverse cardiovascular events when diminished [23]. When both the LV and the RV strains are assessed, incremental prognostic information is provided. In more than 600 patients admitted for acute HF with a median follow-up of 427 days, the inclusion of RV-GLS to conventional echocardiographic measures markedly improved prognostic significance. The patients with worse RV-GLS function experienced unfavorable outcomes [24]. In another study of 171 patients with LV EF < 35%, worse RV strain had an additive predective value. In fact, after adjusting for age, LVEF, RVs, E/e septal, and RA volume index, an absolute value < 14.8% reliably predicted adverse outcomes [73].

Although few studies have examined the prognostic importance of RV systolic dysfunction in patients with HFpEF, it is important to emphasize that the prevalence of this condition ranges from 37% to 19%, and is considered a poor prognostic by multiple mechanisms not yet completely understood [74]. In a study of 183 HFpEF patients, RV-GLS combined with other parameters, such as FAC, TAPSE, and S, was superior in predicting poor outcomes, using an absolute value cutoff <17.5% [75].

Finally, as we already described the pathognomonic apical sparing in cardiac amyloidosis, the analysis of RV strain seems to contain more insights into the mechanisms and the clinical manifestations in these patients. More specifically, the RV-free wall strain correlates with the functional status of amyloid patients, because amyloid deposits infiltrate the RV in later stages, leading to progressive terminal HF [76]. We have shown that both RV strain and RV-GLS are useful for evaluating RV function in patients with HF; however, the former has superior prognostic value, since RV-GLS is influenced by LV dysfunction due to the presence of the interventricular septum [77].

## 3. Conclusions

The objective of this clinical review was to analyze the strengths and weaknesses of GLS in the evaluation of patients with HF. We can consider whether 2D LVEF should be replaced by LV-GLS; the latter, provides pivotal information on prognosis and LV function, but should be intended as complementary. Every new development in diagnostic tools is often time-consuming, and this expenditure should be justified in an improvement of diagnostic and prognostic definitions in clinical practice. The study of LV-GLS in echocardiography proved to have a robust justification in HFpEF patients, to identify pathological versus physiological LV hypertrophy in the initial form of cardiac amyloidosis, and following a cancer patient who is at risk of cardiotoxicity due to chemotherapy.

The study of LA or RV strain seems to be a very promising field for clinical research, and further studies are necessary to translate these results into clinical practice.

In addition, several recent studies have shown that changes in LV and LA strain over time reflect the response to therapy, suggesting their potential value in guiding the optimal management of patients with HF [78]. Finally, the added diagnostic and prognostic values of these methods are summarized in Table 1.

Given all these issues, we may wonder why these approaches are not routinely implemented in our clinical practice: as demonstrated, there are some important limitations to be aware of. Despite providing promising data, these methods are still considered time-consuming, have known limitations and a lack of standard in large studies: a summary of all potential limitations is presented in Table 2.

In conclusion, we are moving in the right direction, improving the prognostic and diagnostic role of the echocardiography with complementary methods, as previously stated. Our hope is that in the future, the accuracy will be improved by technological development, allowing a prevailing use of the method with an accepted standard validation.

## Figures and Tables

**Table 1:** Diagnostic and prognostic implication of GLS.

Diagnostic	Prognostic
improved differentiation between physiological (athlete’s heart, GLS median value 20%) and pathological characteristic improved early diagnosis of cardiac amyloid recognition of early subclinical myocardial injury better evaluation of the LV filling pressure (LA strain < 18%) better assessment of RV performance	association between absolute low GLS and outcomes in HFpEF (<12.8%) and HFrEF (<6.4%) absolute low GLS (<16%) is a predictor of LVEF deterioration after adjusting for other important prognostic markers, association between absolute low RV-GLS in HFrEF (<14.8%), in HFpEF (<17.5%) and outcomes high PSD (>67 msec) is associated to arrhythmic event reduced LA reservoir is associated with poor exercise capacity (<23%)

GLS indicates Global Longitudinal Strain; HFpEF, heart failure with preserved ejection fraction; HFrEF, heart failure with preserved ejection fraction; LA, left atrial; LV, left ventricle; PSD, peak strain dispersion RV, right ventricle.

**Table 2:** Main limitations of strain analysis in LV, RV and LA function.

LV-GLS	LA Strain
Geometric assumption Angle dependent Acoustic window Manual adjustments Large-scale studies are needed to validate the findings in order to have an accepted standard validation Inappropriate measuresdue to imprecise customization or inaccurate ROI	Lack of universally accepted standard validation Preload dependent Manual adjustments Relatively small prevalence of LA strain dysfunction in patients suffering from HFpEF with high LA pressure. Large-scale studies are needed to validate the findings in order to have an accepted standard validation

GLS indicates Global Longitudinal Strain; HFpEF, heart failure with preserved ejection fraction; LA, left atrial; LV, left ventricle; ROI, region of interest.

## Data Availability

Not applicable.
